# A Case of John Cunningham Virus Induced Rhombencephalitis after Rituximab Therapy for Idiopathic Thrombocytopenic Purpura

**DOI:** 10.1155/2021/5525053

**Published:** 2021-06-15

**Authors:** Silpita Katragadda, Varshaa Koneru, Genevieve Devany, Aaron S. DeWitt, Vasudev H. Tati

**Affiliations:** Baton Rouge General Internal Medicine Residency Program, Baton Rouge General Medical Center, Baton Rouge, LA 70809, USA

## Abstract

**Background:**

John Cunningham virus (JCV) is known to cause progressive multifocal leukoencephalopathy (PML) in immuno-compromised patients due to lytic infection of oligodendrocytes and astrocytes. Rarely, it may also present as granule cell neuronopathy (GCN), leading to degeneration of cerebellar granule cell neurons. It is described in patients with underlying conditions or medication contributing to immune compromise. *Case Presentation*. A 73-year-old man presented with ataxia and difficulty in speech which began 3 months after initiation of treatment for idiopathic thrombocytopenic purpura with rituximab. Neurological examination was significant for torsional nystagmus, motor aphasia, right-sided dysmetria, and dysdiadochokinesia with gait ataxia. Magnetic resonance imaging (MRI) showed right cerebellar lesion and cerebrospinal fluid (CSF) polymerase chain reaction (PCR) was positive for JC virus.

**Conclusion:**

The diagnosis of JC virus-related cerebellar disease can be missed, due to the subacute to chronic onset and challenges in detection. Clinicians should have a high degree of suspicion for development of these symptoms, even a few months after initiation of immune-modulatory therapy because the progression and outcomes can be disastrous.

## 1. Introduction

JC virus (JCV) is most commonly known to cause progressive multifocal leukoencephalopathy (PML) in immuno-compromised patients. Granule cell neuronopathy (GCN) is a rare manifestation of the JC virus that affects cerebellar granule cell neurons, caused by a mutation in the viral protein 1 (VP1) gene that results in changes in cell tropism [1]. Here, we describe a case of JCV-related rhomboencephalitis that manifested a few months after initiation of rituximab for idiopathic thrombocytopenic purpura (ITP).

## 2. Case Presentation

A 73-year-old man, who was diagnosed with non-Hodgkin's mantle cell lymphoma, in remission following chemotherapy in 1998, presented in September 2018 with motor aphasia, right upper limb dysmetria, and ataxia. He had a chronic history of idiopathic thrombocytopenic purpura, which was being treated with romiplostim infusions and oral corticosteroids. In June 2018, he developed worsening of idiopathic thrombocytopenic purpura (ITP) and was initiated on weekly rituximab. He received 8 infusions of rituximab. The neurologic exam was notable for torsional nystagmus, slurred speech with intact comprehension, dysmetria on right finger to nose test, and dysdiadochokinesia with gait ataxia. Cognition, strength, and sensation were intact. The biceps, brachioradialis, triceps, and patellar reflexes were 3/4 bilaterally and Achilles reflexes were 2/4 bilaterally. Plantar reflexes were flexor bilaterally.

**Figure 1:**
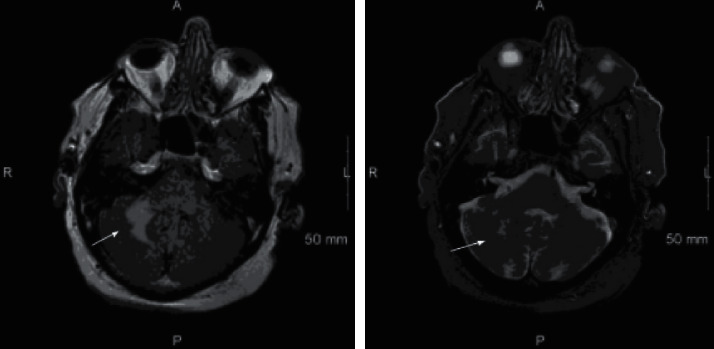
T2 and FLAIR magnetic resonance imaging (MRI) showing a hyperintense lesion without mass effect in the right cerebellar hemisphere.

**Figure 2:**
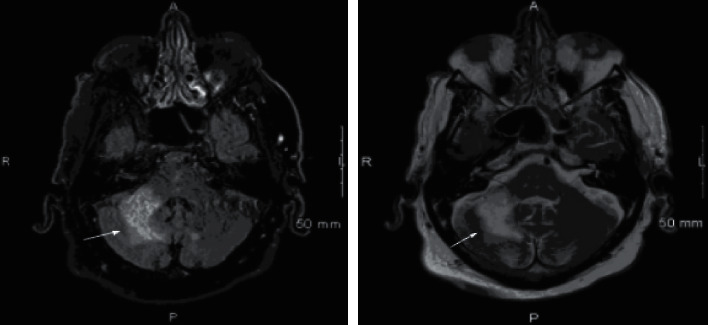
MRI showing interval increase in size of T2 and FLAIR hyperintense infiltrative signal intensity in the right middle cerebellar peduncle and right cerebellar hemisphere as well as a small new focus of FLAIR hyperintense signal intensity in the left cerebellar hemisphere parasagittal to midline.

Magnetic resonance imaging (MRI) demonstrated a hyperintense lesion without mass effect in the right cerebellar hemisphere with no supra tentorial pathology (Figure 1). Extensive workup for etiology of the MRI lesion was negative for vascular, malignant, degenerative, autoimmune, or paraneoplastic etiology. There were no apparent diffusion coefficient (ADC) changes on MRI and the location and shape of the lesions did not fit into a classic vascular distribution. EEG was indicative of a focal right hemisphere central nervous system dysfunction, maximally right fronto-temporal region. HIV, ANA, ceruloplasmin, and serum copper were normal with borderline elevated ACE. Workup for infection with CSF analysis showed lymphocytic pleocytosis with elevated protein, negative cultures, and cytology. CSF was also negative for EBV, HSV, VDRL, West Nile virus, CMV, cryptococcal antigens, and oligo-clonal bands with elevated myelin basic protein. He was given empiric IV steroids in the outpatient setting and MR angiogram was negative for evidence of vasculitis. The patient developed worsening of coordination symptoms with new onset binocular diplopia and partial right third cranial nerve palsy. Repeat MRI showed interval increase in size of T2 and FLAIR hyperintense infiltrative signal intensity in the right middle cerebellar peduncle and right cerebellar hemisphere as well as a small new focus of FLAIR hyperintense signal intensity in the left cerebellar hemisphere parasagittal to midline without enhancement, mass effect, or associated restricted diffusion (Figure 2).

MRI spectroscopy done in October 2018, showed elevation of choline peaks and depression of N-acetyl aspartate (NAA) peaks with mildly prominent lipid peaks, consistent with a cellular process. In November 2018, CSF analysis for JC virus PCR was positive, consistent with JC virus-related rhombencephalitis. Brain biopsy was deferred due to high risk of complications related to location of the lesion.

In December 2018, he was referred to a tertiary care center. On evaluation, he was noted to have progression of lesions on MRI brain with confluent T2/FLAIR hyperintensities in the cerebellar hemispheres, pons, and midbrain compared to the previous scan from November 2018. JC virus PCR in CSF showed 409 copies and JC virus PCR in plasma showed 3200 copies per mL. He was enrolled on a protocol for a phase II study assessing the effect of BK specific cytotoxic T lymphocyte lines (CTLs) generated by ex vivo expansion in patients with BK virus infection and JC virus infection. He received his first infusion of BK specific CTLs in December 2018, to which he had a partial response. Repeat MRI showed decreased patchy enhancement with stable extent of confluent T2 FLAIR signal changes in the cerebellum and pons, suggestive of positive treatment response of progressive multifocal leukoencephalopathy. Repeat testing revealed less than 72 copies per ml of JC virus in CSF and 2100 copies per mL of JC virus in plasma. He tested negative for BK virus in urine and plasma. Second BK specific CTL infusion was given in January 2019.

The patient had multiple emergency room visits and hospital admissions for influenza A and urinary tract infections. He developed progressive weakness and falls and required inpatient rehabilitation to recuperate. Repeat MRI showed increased confluent T2/FLAIR hyperintensities in the cerebellar hemispheres, pons, and midbrain compared to the previous scan. Repeat quantitative JC virus levels in CSF showed an increase (267 copies per mL) compared to prior value, JC virus in plasma was 2000 copies per mL. Given his rapid decline, the family opted to invoke hospice care and was taken off the study in late February 2019.

## 3. Discussion

Polyomavirus JC (JCV) has a sero-prevalence of 50–86% among healthy adults, but remains dormant in immunocompetent persons. Conditions of immunosuppression such as AIDS [2], organ transplant recipients [3], autoimmune diseases treated by immuno-modulatory medications [4, 5], and hematological malignancies [6] tend to cause reactivation of the endemic virus. Clinical presentation can be subacute to chronic symptoms with nonspecific findings on CSF and imaging. The most common manifestation of JCV infection is focal demyelination of the central nervous system called progressive multifocal leukoencephalopathy caused by lytic infection of oligodendrocytes and astrocytes [7].

GCN is shown to exhibit cell tropism. A study of four patients and review of literature of ten cases described variable changes in VP1 structures and a JCV GCN variant of the virus [1]. Another study compared full sequences of JCV isolates from a classic PML lesion and that of a GCN variant. A specific gene deletion was noted on the VP1 gene of the JCV GCN variant. This mutation may explain the predilection of the virus to favor replication in granule cell neurons [8]. GCN can occur alone or in the background of PML, with incoordination, dysarthria and ataxia, and neuroimaging showing atrophy and white matter lesions in the cerebellum [9].

In a review of literature looking at PML after rituximab, median time from the last rituximab dose to PML diagnosis was 5.5 months. Median time to death after PML diagnosis was 2.0 months. The case-fatality rate was 90% [4]. A case of GCN following treatment with rituximab has been described in a case report in which a patient on semi-annual rituximab maintenance therapy developed cerebellar symptoms, five years after frequency was increased to monthly [10].

The PCR is a fast, reliable, and conveniently performed assay. In one study done in 84 HIV negative patients, JC virus was detected by a nested PCR in 6 patients (7%), contributing to confirmation of PML in the background of immunosuppressive conditions. The specificity of the PCR was confirmed by a clinical follow-up study which showed full agreement between the detection of JCV DNA in CSF and clinically manifested PML [11]. The sensitivity and specificity of this PCR were 74 and 95.8%, respectively, while the positive and negative predictive values were 89.5 and 88.5%, respectively [12].

Most conventional DNA PCR tests have been shown to have a cut-off of >50 copies per mL. The sensitivity of the test is not well established because the prevalence of the disease is low. It is important to maintain a high index of clinical suspicion in cases with progressive CNS degeneration with underlying immunosuppression, despite negative PCR. In such cases, ultrasensitive PCR can be used to detect JC virus as it has a lower threshold of >10 DNA copies per mL.

Brain biopsy is the gold standard for diagnosis of GCN, but high risk location of the lesion and hematological contraindications may limit feasibility. On imaging, GCN may present with heterogeneous or nonspecific features. A case series and review of literature described the most common feature to be cerebellar atrophy, which often occurs in combination with white matter changes in the cerebellum and brainstem [13]. The typical imaging finding was not present in our case and biopsy was deferred due to high risk location. Nevertheless, there were clinical signs that correlated with cerebellar involvement along with lesion on imaging. We conclude that this may be an atypical presentation of GCN, the alternate explanation being a presentation of PML alone.

There are no clear studies descriptive of the natural history of the disease. Some case reports have described improvement and stalling of disease progression after stopping the offending agent and recovery of the immune system [9]. Other reports show rapid progressive deterioration has been reported within 17 months (untreated), another despite stopping chemotherapy, eventually culminating in death [10].

## 4. Conclusion

Causation cannot be clearly established between chemotherapy and JC virus leading to GCN in view of underlying lymphoma, but the onset of symptoms in the case described is temporally associated with initiation of rituximab. The clinical presentation can be confounding, with vague symptoms and subacute to chronic onset and noncontributory imaging and CSF findings. So, clinical vigilance to anticipate complications is important while dealing with these patients. Further research is needed to quantify risk of developing PML in patients being initiated on immunosuppressive therapy.
